# Regulation of tNOX expression through the ROS-p53-POU3F2 axis contributes to cellular responses against oxaliplatin in human colon cancer cells

**DOI:** 10.1186/s13046-018-0837-9

**Published:** 2018-07-20

**Authors:** Huei-Yu Chen, Atikul Islam, Tien-Ming Yuan, Shi-Wen Chen, Pei-Fen Liu, Pin Ju Chueh

**Affiliations:** 1 Institute of Biomedical Sciences, National Chung Hsing University, Taichung, 40227 Taiwan; 2 Department of Surgery, Feng-Yuan Hospital, Ministry of Health and Welfare, Taichung, 42055 Taiwan; 3 DDepartment of Food Science and Biotechnology, National Chung Hsing University, 145 Xingda Rd., South Dist, Taichung City, 40227 Taiwan; 4 Graduate Institute of Basic Medicine, China Medical University, Taichung, 40402 Taiwan; 5 Department of Medical Research, China Medical University Hospital, Taichung, 40402 Taiwan; 6 Department of Biotechnology, Asia University, Taichung, 41354 Taiwan

**Keywords:** Apoptosis, Oxaliplatin, p53, Tumor-associated NADH oxidase (tNOX or ENOX2), Sirtuin 1 (SIRT1), ROS

## Abstract

**Background:**

Oxaliplatin belongs to the platinum-based drug family and has shown promise in treating cancer by binding to DNA to induce cytotoxicity. However, individual patients show diverse therapeutic responses toward oxaliplatin due to yet-unknown underlying mechanisms. We recently established that oxaliplatin also exert its anti-cancer activity in gastric cancer cell lines by targeting tumor-associated NADH oxidase (tNOX), attenuate NAD^+^ generation and reduce NAD^+^-dependent sirtuin 1 (SIRT1) deacetylase activity, which in turn enhances p53 acetylation and apoptosis.

**Methods:**

In this study, differential cellular outcomes in response to oxaliplatin exposure of p53-wild-type versus p53-null HCT116 human colon cancer cells were examined. Cell growth profile was determined by cell impedance measurements and apoptosis was analyzed by flow cytometry. The engagement between oxaliplatin and tNOX protein was studied by cellular thermal shift assay. Furthermore, western blot analysis revealed that p53 was important in regulating tNOX expression in these cell lines.

**Results:**

In p53-wild-type cells, we found that oxaliplatin inhibited cell growth by inducing apoptosis and concurrently down-regulating tNOX at both the transcriptional and translational levels. In p53-null cells, in contrast, oxaliplatin moderately up-regulated tNOX expression and yielded no apoptosis and much less cytotoxicity. Further experiments revealed that in p53-wild-type cells, oxaliplatin enhanced ROS generation and p53 transcriptional activation, leading to down-regulation of the transcriptional factor, POU3F2, which enhances the expression of tNOX. Moreover, the addition of a ROS scavenger reversed the p53 activation, POU3F2 down-regulation, and apoptosis induced by oxaliplatin in p53-wild-type cells. In the p53-null line, on the other hand, oxaliplatin treatment triggered less ROS generation and no p53 protein, such that POU3F2 and tNOX were not down-regulated and oxaliplatin-mediated cytotoxicity was attenuated.

**Conclusion:**

Our results show that oxaliplatin mediates differential cellular responses in colon cancer cells depending on their p53 status, and demonstrate that the ROS-p53 axis is important for regulating POU3F2 and its downstream target, tNOX. Notably, the depletion of tNOX sensitizes p53-null cells to both spontaneous and oxaliplatin-induced apoptosis. Our work thus clearly shows a scenario in which targeting of tNOX may be a potential strategy for cancer therapy in a p53-inactivated system.

## Background

The newest numbers indicate that colorectal cancer (CRC) remains the third most commonly diagnosed cancer, and 50,260 deaths from this disease were predicted in the United States for 2017 [1]. Despite extensive efforts to identify a better therapeutic strategy, chemotherapeutic drugs continue to be widely used to treat CRC, especially at its advanced stages. The utilized drugs include, oxaliplatin, which belongs to the third generation of platinum-based compounds; these drugs bind and form DNA adducts that interfere with DNA replication to initiate DNA damage and apoptosis [2, 3]. Unfortunately, the anti-cancer efficacy of oxaliplatin often hindered by tumor-related resistance, which is frequently associated with changes in cellular detoxification and transportation, DNA damage and repair, and cell death [4]. Thus, it is not surprising that p53, which is a functional tumor suppressor that plays paramount roles in regulating cell cycle progression, DNA repair, and apoptosis, is associated with the susceptibility of tumors to oxaliplatin-based therapy [5–8]. p53 is inactive or mutated in many CRC patients; this leads to an acquired resistance that considerably limits the effectiveness of therapeutic drugs for cancer management. To address this, researchers are seeking to identify and further target additional factors that contribute to drug resistance in the hopes of designing new therapeutic strategies.

We recently examined the effects of oxaliplatin in three gastric cancer cell lines that differed in their p53 status, and found that oxaliplatin-mediated cytotoxicity and apoptosis induction were very dissimilar among them [9]. Interestingly, the responsiveness toward oxaliplatin in these cell lines corresponded to both their p53 functionality and the expression level of tumor-associated NADH oxidase (tNOX) [9]. tNOX catalyzes the conversion of reduced NADH to oxidized NAD^+^; it universally expressed in a wide range of transformed/cancerous lines and is positively correlated with cell growth ability [10–13]. Numerous anticancer drugs (oxaliplatin, capsaicin, doxorubicin and its derivatives, etc.) have been shown to trigger tNOX down-regulation and concurrent apoptosis [9, 14–18]. Our recent work showed that oxaliplatin diminishes the ratio between tNOX-catalyzed intracellular NAD^+^ and NADH, and thereby lessens NAD^+^-dependent SIRT1 deacetylase activity, resulting in p53 acetylation and apoptosis induction in p53-wild-type gastric cancer cells. Conversely, oxaliplatin had much smaller inhibitory effects on tNOX activity/expression and triggered little apoptosis in p53-mutated cells [9]. Furthermore, forced depletion of tNOX by RNA interference in these resistant p53-mutated cells augmented their spontaneous apoptosis, increased their drug sensitivity, and diminished their cell growth [9]. These findings and those of other studies strongly support the notion that tNOX may be a potential therapeutic drug target [14, 15, 18–21]. However, it was unclear whether p53 participates in governing anticancer-drug-induced inhibition of tNOX and subsequent apoptosis of cancer cells.

Here, we focused on the p53 status and regulation of tNOX expression in HCT116 human colon cancer p53-wild-type and –null cells and sought to decipher the molecular basis underlying their differential cellular responses to oxaliplatin. We found that oxaliplatin mediated very different outcomes in these cells depending on their p53 functionality, ranging from increasing stress tolerance to apoptosis induction, and that these effects were associated with distinct transcriptional and translational regulations of tNOX.

## Methods

### Materials

Fetal bovine serum (FBS) and penicillin/streptomycin were obtained from Gibco/BRL Life Technologies (Grand Island, NY, USA). The anti-Bak, anti-Bax, anti-Bcl-2, anti-PARP, anti-p53, anti-phospho-p53, anti-acetyl-p53, and anti-SIRT1 antibodies were purchased from Cell Signaling Technology, Inc. (Beverly, MA, USA). The anti-survivin antibody was purchased from Santa Cruz Biotechnology, Inc. (Santa Cruz, CA, USA). The anti-POU3F2 antibody was from GeneTex, Inc. (Irvine, CA, USA). The anti-β-actin antibody was from Millipore Corp. (Temecula, CA, USA). The antisera to tNOX used in Western blot analyses were generated as described previously [22]. The anti-mouse and anti-rabbit IgG antibodies and other chemicals were purchased from Sigma Chemical Company (St. Louis, MO, USA) unless otherwise specified.

### Cell culture and transfection

HCT116 (human colorectal cancer) wild-type (The Bioresource Collection and Research Center, BCRC, Hsinchu, Taiwan) and p53^−/−^ (from Horizon Discovery, Cambridge, UK) cells were grown in McCoy’s 5A medium. AGS (gastric adenocarcinoma, p53 wild-type) and TMK-1 (from a poorly differentiated adenocarcinoma, mutant p53) human stomach cancer cells, which were grown in RPMI-1640, were kindly provided by Dr. Chun-Ying Wu (Department of Gastroenterology, Taichung Veterans General Hospital, Taiwan). SAS (tongue squamous carcinoma, p53 functional) and HSC-3 (oral squamous carcinoma, p53 inactive) cells, which were grown in DMEM, were kindly provided by Dr. Yuen-Chun Li (Department of Biomedical Sciences, Chung Shan Medical University, Taiwan). All media were supplemented with 10% fetal bovine serum, 100 units/mL penicillin and 50 μg/mL streptomycin. The cells were grown at 37°C in a humidified atmosphere of 5% CO_2_ in air, with replacement of the medium every 2–3 days. The experimental groups were treated with different concentrations of oxaliplatin dissolved in ddH_2_O, and the controls were treated with the same volume of ddH_2_O.

ON-TARGETplus tNOX (ENOX2) siRNA and negative control siRNA were purchased from Thermo Scientific, Inc. (Grand Island, NY) and the POU3F2-specific and negative control RNAi were constructed at the RNAi core facility of Academic Sinica (Taipei, Taiwan, ROC). Briefly, cells were seeded in 10-cm dishes, allowed to attach overnight, and then transfected with tNOX/POU3F2 siRNA or control siRNA using the Lipofectamin RNAiMAX Reagent (Gibco/BRL Life Technologies) according to the manufacturer’s instructions.

### Plasmid constructs and luciferase assay

The full protein-encoding sequence of the *POU3F2* gene was amplified from human cDNA and the generated PCR products were cloned into the pCDNA3.1/Myc_His (+)A vector, and the obtained construct was used for POU3F2 overexpression experiments.

Fourteen-hundred base pairs of the 5’-flanking DNA sequence of the *tNOX* gene were PCR amplified from the genomic DNA of HCT116 cells. The PCR products were subcloned into the pGL3-Basic luciferase reporter vector (Promega, Madison, WI, USA) to generate the pGL-1.4 kb construct for reporter assays. The reporter vectors plus the POU3F2 expression plasmid or empty vector were co-transfected into HCT116 p53 wild type cells using Lipofectamine 2000 (Promega) according to the manufacturer’s instructions. Cells were harvested 48 h after transfection, and luciferase activity was measured using the Dual-Luciferase Reporter Assay System (Promega) according to the manufacturer’s instructions.

### Continuous monitoring of cell impedance

For continuous monitoring of changes in cell growth, cells (7.5 × 10^3^ cells/well) were seeded onto E-plates and incubated for 30 min at room temperature. The E-plates were placed onto a Real-Time Cell Analysis (RTCA) station (Roche, Germany) and the cells were grown overnight before being exposed to oxaliplatin or ddH_2_O. Cell impedance was measured every hour for a total of 72 h, as previously described [23], and was defined by the cell index (CI) = (Z_i_ − Z_0_) [Ohm]/15[Ohm], where Z_0_ is the background resistance and Z_i_ is the resistance at an individual time point. A normalized CI was determined as the CI at a certain time point (CI_ti_) divided by that at the normalization time point (CI_nml_time_).

### Apoptosis determination

Apoptosis was measured using an Annexin V-FITC apoptosis detection kit (BD Pharmingen, San Jose, CA, USA). Cells cultured in 6-cm dishes were trypsinized, collected by centrifugation, washed, resuspended in 1× binding buffer, and stained with Annexin V-FITC, as recommended by the manufacturer. Cells were also stained with propidium iodide (PI) to detect necrosis or late apoptosis. The distributions of viable (FITC/PI double-negative), early apoptotic (FITC-positive), late apoptotic (FITC/PI double-positive), and necrotic (PI-positive/FITC-negative) cells were analyzed using a FC500 flow cytometer (Beckman Coulter, Inc. Indianapolis, IN). The results are expressed as a percentage of total cells.

### Cellular thermal shift assay (CETSA)

Engagement between oxaliplatin and tNOX in cells was analyzed by CETSA. Samples were prepared from control cells and those exposed to the drug. For each set, 2 × 10^7^ cells were seeded in a 10-cm cultured dish. After 24 h of culture, the cells were pretreated with 10 μM MG132 for 1 h, washed with PBS, treated with trypsin, and collected. Samples were centrifuged at 12,000 rpm for 2 min at room temperature, the pellets were gently resuspended with 1 mL of PBS, and the samples were centrifuged at 7500 rpm for 3 min at room temperature. The pellets were resuspended with 1 mL of PBS containing 20 mM Tris-HCl pH 7.4, 100 mM NaCl, 5 mM EDTA, 2 mM phenylmethylsulfonyl fluoride (PMSF), 10 ng/ml leupeptin, and 10 μg/ml aprotinin. The samples were transferred to Eppendorf tubes, subjected to three freeze-thaw cycles; for each cycle, they were exposed to liquid nitrogen for 3 min, placed in a heating block at 25 °C for 3 min, and vortexed briefly. The samples were then centrifuged at 12,000 rpm for 30 min at 4 °C, and the supernatants were transferred to new Eppendorf tubes. For the experimental sample set, oxaliplatin was added to a final concentration of 100 μM; for the control sample set, the same volume of vesicle solvent was added. The samples were heated at 37 °C for 1 h and dispensed to 100 μl aliquots. Pairs consisting of one control aliquot and one from experimental aliquots were heated at 40 °C, 43 °C, 46 °C, 49 °C, 52 °C, 55 °C, 58 °C, 61 °C, or 67 °C for 3 min. Finally, the samples were placed on ice and subjected to Western blot analysis using antisera to tNOX [22].

### Measurement of the intracellular NAD^+^/NADH ratio

The oxidized and reduced forms of intracellular NAD were determined using an NADH/NAD Quantification Kit (BioVision Inc. Milpitas, CA, USA), as described by the manufacturer. Briefly, 2 × 10^5^ cells were washed with cold PBS, pelleted, and extracted by two freeze/thaw cycles with 400 μl of NADH/NAD^+^ extraction buffer. The samples were vortexed and centrifuged at 14,000 rpm for 5 min. The extracted NADH/NAD^+^ supernatant (200 μl) was transferred to a microcentrifuge tube, heated to 60 °C for 30 min (to decompose NAD^+^ but not NADH), and then placed on ice. The samples were then centrifuged and transferred to a multiwell-plate. Standards and a NAD^+^ cycling mix were prepared according to the manufacturer’s protocol. The reaction mix (100 μl) was distributed to each well containing NADH standards and samples, and the plates were incubated at room temperature for 5 min to convert NAD^+^ to NADH. The provided NADH developer solution was added to each well, and plates were incubated at room temperature for 15 or 30 min. The reaction was stopped with 10 μl of stop solution per well, and absorbance was measured at 450 nm.

### Reverse transcriptase-polymerase chain reaction (RT-PCR)

Total RNA from gastric cancer cells was isolated using the TRIzol reagent (Gibco, Carlsbad, CA, USA). First strand cDNA was synthesized from 1 μg of total RNA using Superscript II (Life Technologies, Rockville, MD, USA). The following primers sets were used for PCR amplifications: tNOX, 5’-GAAGTGTGATGCCGATAACAG -3″ (sense) and 5’-AGTACTAGAGCCCAGGCGAA-3′ (antisense); POU3F2, 5’-TGGGATTTACCCAAGCGGAC -3″ (sense) and 5’-TGTGGTGGAGTGTCCCTACT-3′ (antisense);and β-actin, 5’-ACTCACCTTGGTGGTGCATA-3′ (sense) and 5’-ACACCTTGATGGGAAAGGTGG-3′ (antisense). The reaction conditions consisted of 30 cycles of 95 °C for 30 s, 55 °C for 30 s, and 72 °C for 1 min, followed by a final extension of 5 min at 72 °C. The obtained PCR products were resolved by 1.4% agarose gels electrophoresis and visualized by ethidium bromide staining.

### Measurement of reactive oxygen species (ROS)

Oxidative stress was determined by measuring the level of hydrogen peroxide (H_2_O_2_) generated in the cells, as assessed by 5-(6)-carboxy-2′,7′-dichlorodihydrofluorescein diacetate (carboxy-H_2_DCFDA) staining. The nonpolar, nonionic H_2_-DCFDA is cell permeable and is hydrolyzed to nonfluorescent H_2_-DCF by intracellular esterases. In the presence of peroxide, H_2_-DCF is rapidly oxidized to highly fluorescent DCF. In brief, at the end of oxaliplatin treatment, cells (2 × 10^5^) were washed with PBS and incubated with 5 μM H_2_DCFDA in DMSO for 30 min. The cells were then collected by trypsinization and centrifugation, washed with PBS, centrifuged at 200×g for 5 min and analyzed immediately using a Beckman Coulter FC500 flow cytometer.

### Western blot analysis

Cell extracts were prepared in lysis buffer containing 20 mM Tris-HCl pH 7.4, 100 mM NaCl, 5 mM EDTA, 2 mM PMSF, 10 ng/ml leupeptin, and 10 μg/ml aprotinin. Equal amounts of extracted proteins (40 μg) were resolved by SDS-PAGE and transferred to nitrocellulose membranes (Schleicher & Schuell, Keene, NH, USA). The membranes were blocked with nonfat milk solution for 1 h, and then washed and probed with the appropriate primary antibody. The membranes were rinsed with Tris-buffered saline containing 0.1% Tween 20, incubated with horseradish peroxidase-conjugated secondary antibody for 1 h, rinsed again, and developed using enhanced chemiluminescence (ECL) reagents (Amersham Biosciences, Piscataway, NJ, USA).

### Statistics

All data are expressed as the means ± SEs of three or more independent experiments. Between-group comparisons were performed using one-way analysis of variance (ANOVA) followed by an appropriate post-hoc test. A value of *p* < 0.05 was considered to be statistically significant.

## Results

### The ability of oxaliplatin to attenuate growth and induce apoptosis in colon cancer cells depend on the p53 status

**Fig. 1:**
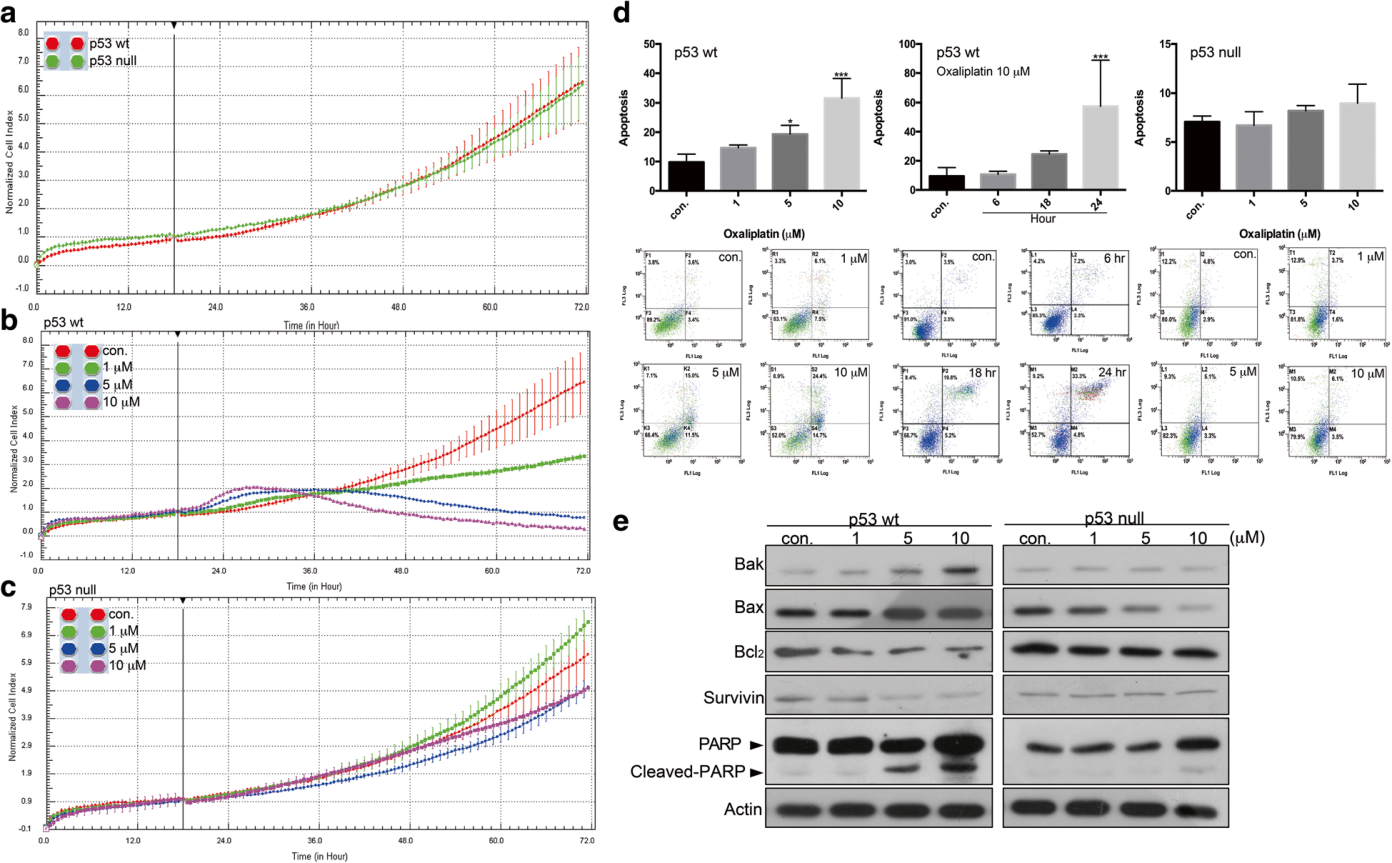
Oxaliplatin suppresses the growth of HCT116 p53-wild-type cells by inducing apoptosis, but no such effect is seen in p53-null cells. A-C, HCT116 cells were treated without (**a**) or with (**b** and **c**) oxaliplatin and cell growth was dynamically monitored using impedance technology. Normalized cell index values measured over 70 h are shown. **d**, HCT116 cells were treated with oxaliplatin or ddH_2_O for different concentrations or time length and the percentage of apoptotic cells was determined by flow cytometry. The results are presented as the percentage of apoptotic cells; the presented values (mean ± SEs) represent three independent experiments performed in at least triplicate (*p < 0.05, ***p < 0.001 for cells treated with oxaliplatin vs. controls). Representative images are shown. **e**, HCT116 cells were treated with oxaliplatin or ddH_2_O for 24 h, and cell lysates were separated by SDS-PAGE and analyzed by Western blotting. β-Actin was used as an internal control. Representative images are shown

We herein utilized p53-wild-type and p53-null (gene knockout) HCT116 cells to study whether the effect of oxaliplatin on colon cancer cells is dependent on the functionality of p53. Monitoring of dynamic cell growth by cell impedance measurements, revealed that the presence of p53 does not affect the growth profile of HCT116 cells, since both lines exhibited similar growth rates over 72 h (Fig. 1a). Oxaliplatin greatly and dose-dependently attenuated the growth of p53-wild-type cells, with an IC_50_ value of 0.65 μM (Fig. 1b). In p53-null cells, in contrast, oxaliplatin did not have any marked inhibitory effect at the tested concentrations; in fact, 1 μM oxaliplatin appeared to stimulate cell growth, and the IC_50_ value was high at 9.67 μM (Fig. 1c). This difference in cytotoxicity was also reflected in the apoptotic populations of oxaliplatin-exposed cells: we observed marked increase in the apoptosis of p53-wild-type cells exposed to oxaliplatin in a dose- and time-dependent fashion, whereas minimal (insignificant) apoptosis was induced in p53-null cells exposed to the same doses of oxaliplatin (Fig. 1d). Protein analysis confirmed that the oxaliplatin-induced apoptosis of p53-wild-type cells was associated with up-regulation of the pro-apoptotic proteins, Bak and Bax, down-regulation of the pro-survival factors, Bcl2 and survivin, and the direct cleavage of PARP by caspase 3 (Fig. 1e, left panel). Most recently, the utilization of a single molecule localization microscopy revealed that densely packed Bak clusters on mitochondria are critical for the induction of apoptosis [24]. It is proposed that oxaliplatin-induced Bak up-regulation in p53-wild-type cells observed in this present study may play a necessary role in apoptosis (Fig. 1e, left panel). Consistent with the lack of marked oxaliplatin-induced apoptosis, however, these protein changes were not observed in p53-null cells treated with oxaliplatin (Fig. 1e, right panel). Interestingly, p53-null cells exposed to oxaliplatin exhibited a Bax down-regulation, associating with little cytotoxicity; such a phenomena is also correlated with colorectal carcinogenesis and drug resistance as reported elsewhere [25].

### Oxaliplatin engages with tNOX protein and inhibits its enzymatic activity independent of p53

**Fig. 2:**
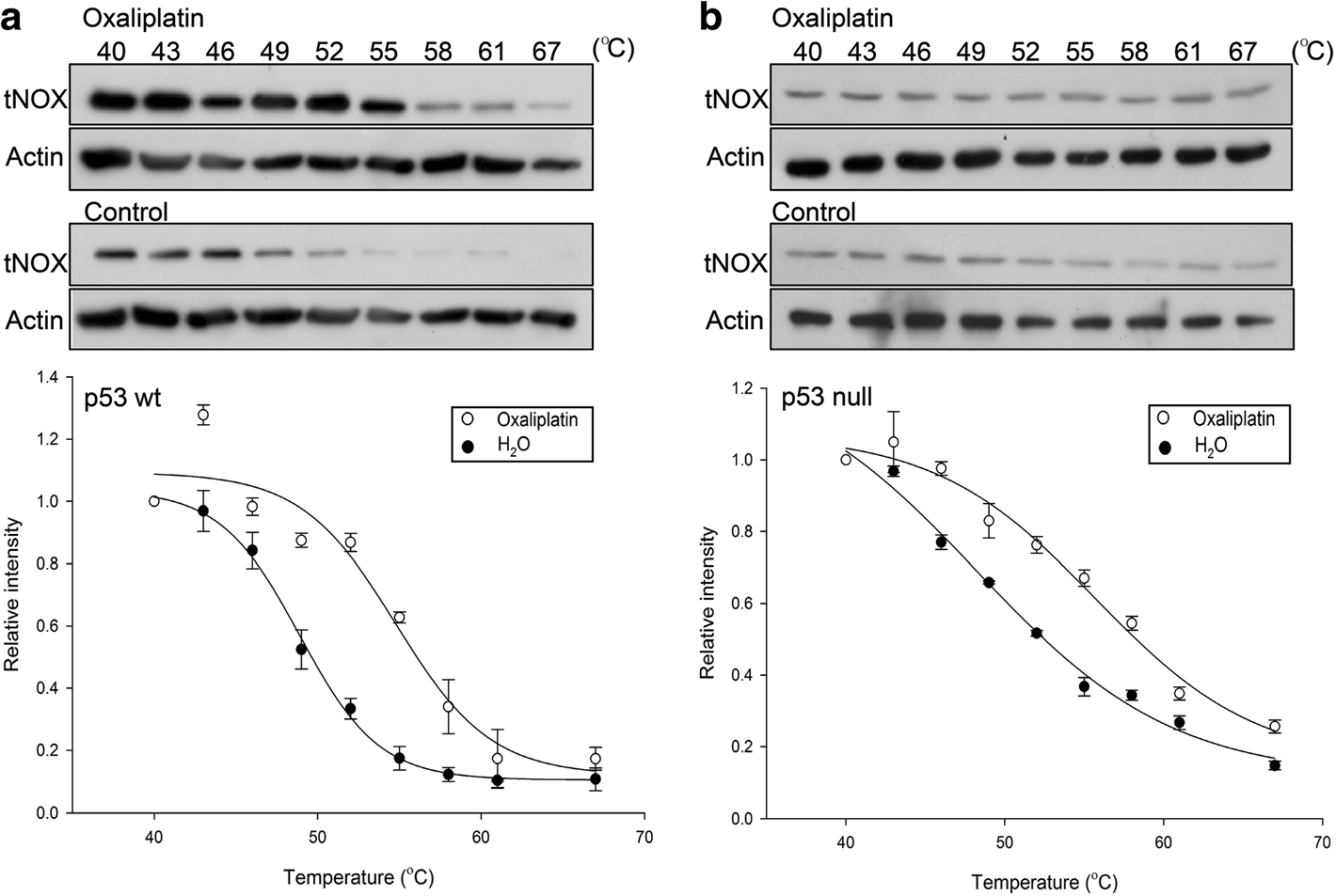
CETSA-based determination of binding between oxaliplatin and tNOX. A, B, CETSA curves of tNOX in p53-wild-type cells (**a**) and p53-null cells (**b**) were determined in the absence and presence of 100 μM oxaliplatin, and cell lysates were separated by SDS-PAGE and analyzed by Western blotting. β-Actin was used as an internal control. Representative images are shown. The band intensities of tNOX were normalized with respect to the intensity at 40 °C. The denaturation midpoints were determined using a standard process

We previously showed that the diverse responsiveness of different stomach cancer lines toward oxaliplatin is reflected in the protein level of tumor-associated NADH oxidase (tNOX) [9]. In the present study, we further investigated whether oxaliplatin engages tNOX to contribute to the induction of the apoptosis in p53-wild-type colon cancer cells. The cellular thermal shift assay (CETSA) is a label-free assay that may be used to study target engagement in cells based on the ability of ligand binding to enhance the stability of target proteins [26, 27]. For CETSA, thermal melting curves are plotted and the melting temperature (*T*_m_; the temperature at which 50% of proteins are unfolded and rapidly precipitated by heat) can be derived. We observed an obvious shift in the thermal melting curves in the oxaliplatin-treated lysates of p53-wild-type cells compared to control lysates. The *T*_m_ value increased from 48.9 °C (control) to 54.8 °C (oxaliplatin-treated), suggesting that oxaliplatin induced thermal stabilization of tNOX using antisera to tNOX (Fig. 2a). In p53-null cells, our results also suggested that oxaliplatin binds to tNOX, with the *T*_m_ values increasing from 48.2 °C (control) to 55.7 °C (oxaliplatin-treated) (Fig. 2b). The addition of oxaliplatin increased in *T*_m_ in both cases, indicating that the drug bound to the tNOX protein. However, the heat-induced unfolding of the thermal melting curves differed between the p53-null and p53-wild-type cells, the latter of which showed a prominent sigmoidal pattern. This suggests that the tNOX-unfolding process might differ in cellular environments with or without p53.

**Fig. 3:**
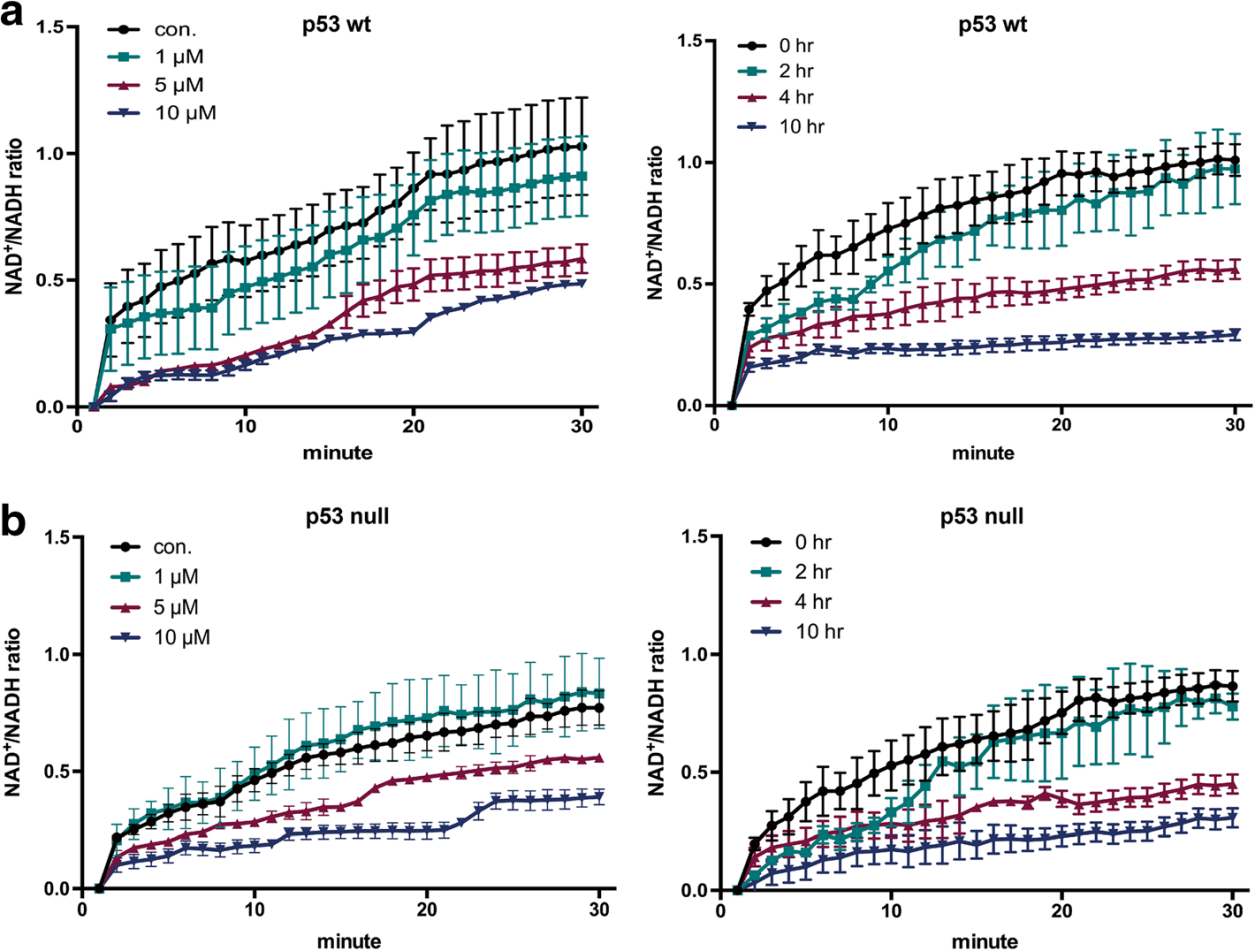
Oxaliplatin dose- and time-dependently reduces the NAD^+^/NADH ratio in HCT116 p53-wild-type (**a**) and p53-null (**b**) cells. Cells were treated with ddH_2_O or oxaliplatin, and the NAD^+^ and NADH levels in cell extracts were quantified based on the optical density at 450 nm, the NAD^+^/NADH ratio was calculated. Values (mean ± SEs) were obtained from at least three independent experiments

As our CETSA results indicated that oxaliplatin does indeed bind tNOX, we next analyzed whether oxaliplatin binding affects the enzymatic ability of tNOX to convert reduced NADH to oxidized NAD^+^ [11, 12]. We found that oxaliplatin concentration- and time-dependently decreased the intracellular NAD^+^/NADH ratio in p53-wild-type cells compared to controls, possibly by attenuating tNOX oxidase activity (Fig. 3a). This supports our previous contention that oxaliplatin binds to tNOX and inhibits its enzymatic activity, which in turn reduces the intracellular NAD^+^/NADH ratio [9]. Unexpectedly, a similar pattern was observed in p53-null cells (Fig. 3b). This suggests that the binding of oxaliplatin to tNOX and its effect on enzymatic activity might not be sufficient to explain the different cellular outcomes observed in these two cell lines.

### Oxaliplatin-induced apoptosis depends on tNOX down-regulation

**Fig. 4:**
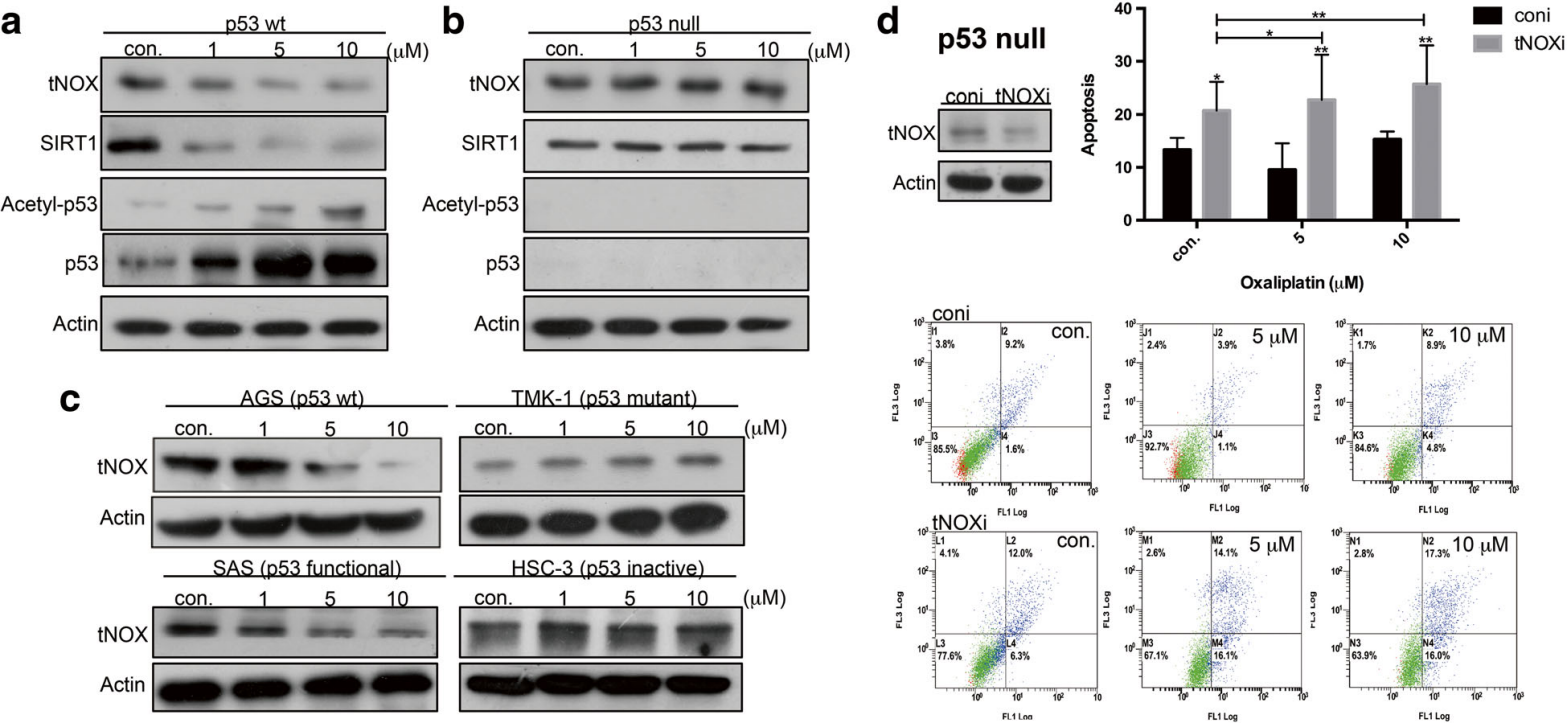
Oxaliplatin reduces tNOX and SIRT1 expression in p53-wild-type cells but not in p53-null or -mutant cells. **a**-**c**, Cell lysates were separated by SDS-PAGE and analyzed by Western blotting. β-Actin was used as an internal control. Representative images are shown. **d**, tNOX was knocked down by RNA interference in p53-null cells. The cells were treated with ddH_2_O or oxaliplatin for 24 h, and the percentage of apoptotic cells was determined by flow cytometry. The presented values (mean ± SEs) represent at least three independent experiments (*p < 0.05 or **p < 0.01 for tNOX-knockdown cells vs. controls). Representative images are shown

To search for another mechanism that might explain the between-line differences, we next examined tNOX expression in cells treated with oxaliplatin. Western blot analysis showed that oxaliplatin dose-dependently attenuated the level of tNOX in p53-wild-type cells, but had little inhibitory effect in p53-null cells (Fig. 4a and b). Given that tNOX catalyzes the oxidation of NADH to NAD^+^, and the latter is a cofactor for sirtuin (SIRT1) activity, we next analyzed the impact of oxaliplatin on SIRT1 activity. Interestingly, the protein level of SIRT1 was attenuated and the acetylation of p53 was increased in p53-wild-type cells, suggesting that SIRT1 deacetylase activity was inhibited in these cells (Fig. 4a). This presence of p53 acetylation/activation in wild-type cells is likely to contribute to the ability of oxaliplatin to induce apoptosis (see Fig. 1). In the p53-null cell line, in contrast, oxaliplatin had no inhibitory effect on tNOX expression and SIRT1 expression remained unchanged (Fig. 4b).

The above findings suggest that the down-regulation of tNOX in p53-wild-type cells may explain the differential cellular responses to oxaliplatin in the tested cell lines. Indeed, we obtained comparable results in oxaliplatin-treated AGS (wild-type p53) and TMK-1 (mutant p53) gastric cancer cells (Fig. 4c), and noted that these findings were consistent with the profound apoptotic activity of oxaliplatin in AGS but not TMK-1 cells [9]. Similarly, oxaliplatin induced tNOX down-regulation in SAS tongue squamous carcinoma cells, which carry an E336X mutant (a stop cordon), but exhibiting wild-type p53 function, whereas tNOX expression remained unchanged upon oxaliplatin exposure in HSC-3 oral squamous carcinoma (p53 inactive) cells (Fig. 3c). To test whether the lack of oxaliplatin-induced apoptosis in p53-null/mutated cells is associated with the stability of tNOX, we performed tNOX-specific RNA interference (RNAi) in p53-null HCT116 cells. Indeed, knockdown of tNOX enhanced spontaneous apoptosis in p53-null cells compared to control cells, regardless of oxaliplatin exposure (Fig. 4d). Moreover, tNOX-depleted p53-null cells treated with 5 or 10 μM oxaliplatin demonstrated evident induction of apoptosis, whereas the drug was not apoptotic in scrambled siRNA-transfected cells (Fig. 4d). Thus, tNOX appears to be essential for cancer cell survival. These converging lines of evidence reveal that tNOX expression and the ultimate cellular response toward oxaliplatin differ markedly in cells with or without functional p53, further suggesting that targeting of tNOX could be an alternative therapeutic strategy in p53-inactivated cancer scenarios.

### Ubiquitination and proteasome degradation contribute to the ability of oxaliplatin to translationally reduce tNOX expression in p53-wild-type cells

**Fig. 5:**
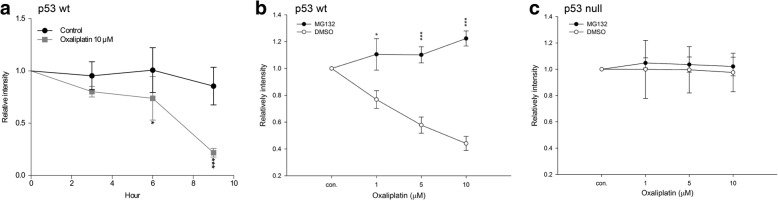
Oxaliplatin decreases tNOX expression in HCT116 p53-wild-type cells but not in p53-null cells. Cells were pretreated with 50 μg/mL CHX (**a**) or MG132 (**b**, **c**) for 30 min and exposed to oxaliplatin (10 μM) for 24 h, and then analyzed for tNOX expression. The protein intensity of tNOX was determined by densitometric analysis and normalized to the relevant β-actin value. The presented results represent at least three independent experiments (*p < 0.05 or ***p < 0.001 for treatments vs. controls)

Given that tNOX expression is important for regulating apoptosis and the down-regulation of tNOX depends on p53 functionality, we examined whether the oxaliplatin-induced down-regulation of tNOX expression in p53-wild-type cells was due to protein degradation. We analyzed protein stability using a cycloheximide-chase assay and found that the half-life of tNOX was markedly decreased in p53-wild-type cells exposed to oxaliplatin for 6 and 9 h (Fig. 5a). Treatment with the proteasome inhibitor, MG132, significantly improved the stability of tNOX in p53-wild-type cells exposed to oxaliplatin (Fig. 5b). As expected, tNOX stability remained largely unchanged in p53-null cells regardless of oxaliplatin treatment, and MG132 treatment did not alter tNOX stability in these cells (Fig. 5c). These findings indicate that proteasome degradation is indeed involved in the oxaliplatin-induced down-regulation of tNOX in p53-wild-type cells but not in p53-null cells.

### ROS-modulated POU3F2 contributes to the oxaliplatin-induced transcriptional modulation of tNOX expression

**Fig. 6:**
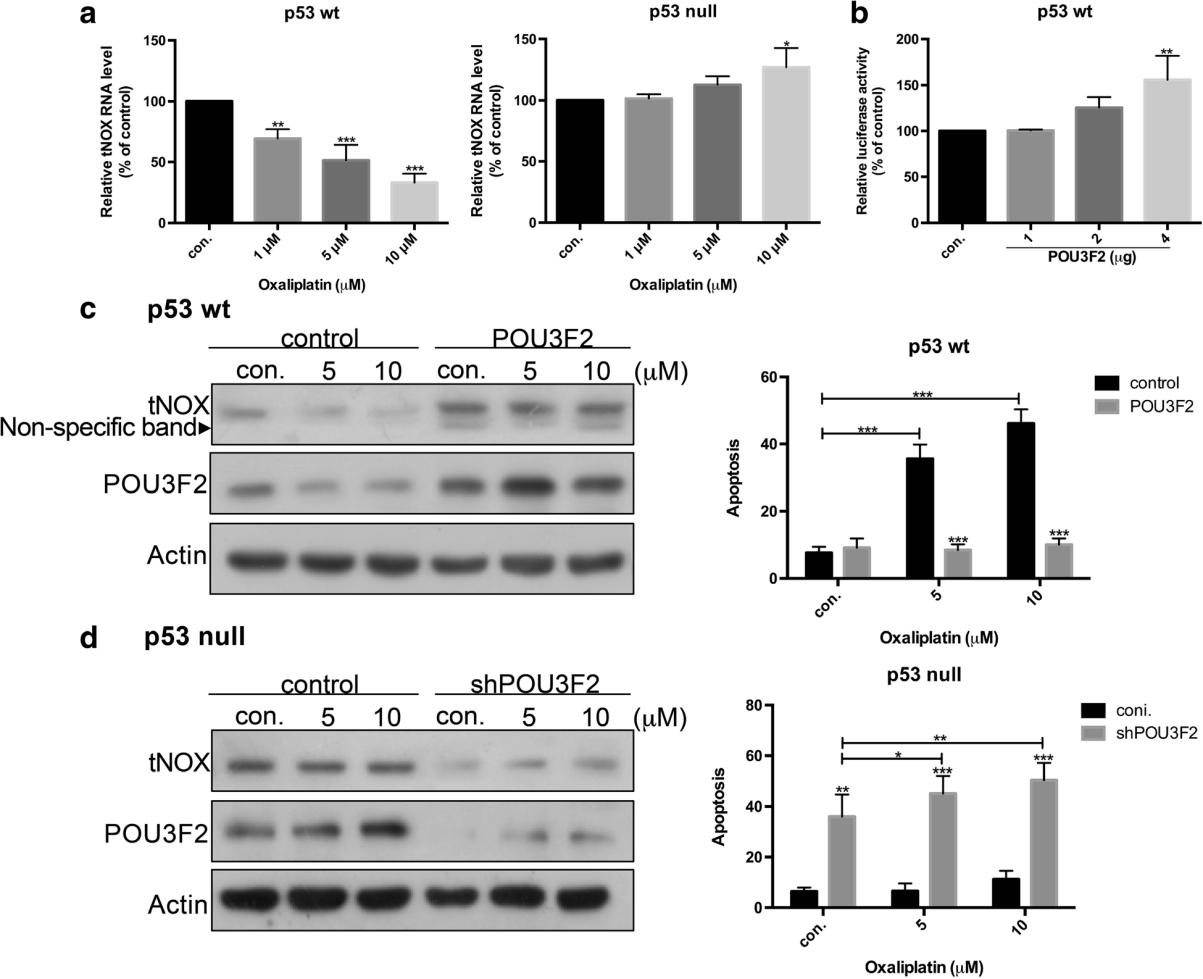
**a**, HCT116 cells were treated with oxaliplatin or ddH_2_O for 24 h, and tNOX mRNA levels were determined by RT-PCR. The presented results represent three independent experiments (**p < 0.01, ***p < 0.001 for cells treated with oxaliplatin vs. controls). **b**, p53-wild-type cells were co-transfected with a reporter construct and a POU3F2 expression vector, and luciferase activities were determined. The presented values (mean ± SD) represent three independent experiments performed in at least triplicate (**p < 0.01 for experimental group vs. control). **c**, p53-wild-type cells were transfected with the POU3F2 expression vector for 24 h and then exposed to oxaliplatin for an additional 24 h. Cell lysates were separated by SDS-PAGE and analyzed by Western blotting. β-Actin was used as an internal control. Representative images are shown. The percentage of apoptotic cells was also determined by flow cytometry; the presented values (mean ± SEs) represent at least three independent experiments (***p < 0.001 for treatments vs. controls). **d**, p53-null cells were transfected with the shPOU3F2 knockdown vector for 24 h and then exposed to oxaliplatin for an additional 24 h. Cell lysates were separated by SDS-PAGE and analyzed by Western blotting. β-Actin was used as an internal control. Representative images are shown. The percentage of apoptotic cells was also determined by flow cytometry; the presented values (mean ± SEs) represent at least three independent experiments (*p < 0.05, **p < 0.01, ***p < 0.001 for treatments vs. controls)

When we examined the effect of oxaliplatin on the transcriptional expression of tNOX, we found that the drug markedly and concentration-dependently reduced tNOX RNA expression in p53-wild-type cells, whereas that in p53-null cells was gradually enhanced by oxaliplatin as the dose increased, reaching a significant level in cells treated with 10 μM oxaliplatin (Fig. 6a). As a previous study showed that the POU domain transcription factor, POU3F2, positively regulates tNOX expression in gastric cancer cells [28], we examined the potential involvement of POU3F2 in this system. The 1.4 kb-5′ promoter region of the tNOX gene was subcloned into the pGL3-basic luciferase reporter vector. We found that co-transfection of HCT116 p53-wild-type cells with the pGL3–1.4 kb promoter and different amounts of a POU3F2-expressing plasmid yielded a concentration-dependent increase in luciferase activity (Fig. 6b), providing a direct link between POU3F2 and tNOX expression. We further found that the oxaliplatin-induced down-regulation of tNOX in p53-wild-type cells was accompanied by attenuated POU3F2 expression; that POU3F2 overexpression up-regulated tNOX expression in these cells; and that oxaliplatin did not induce apoptosis in these POU3F2-overexpressing and tNOX-up-regulated cells (Fig. 6c). In p53-null cells, tNOX expression remained stable following oxaliplatin exposure, but POU3F2 knockdown diminished tNOX expression and was associated with marked apoptosis (Fig. 6d). These findings suggest that POU3F2 is involved in the oxaliplatin-mediated regulation of tNOX and the subsequent cellular responses.

**Fig. 7:**
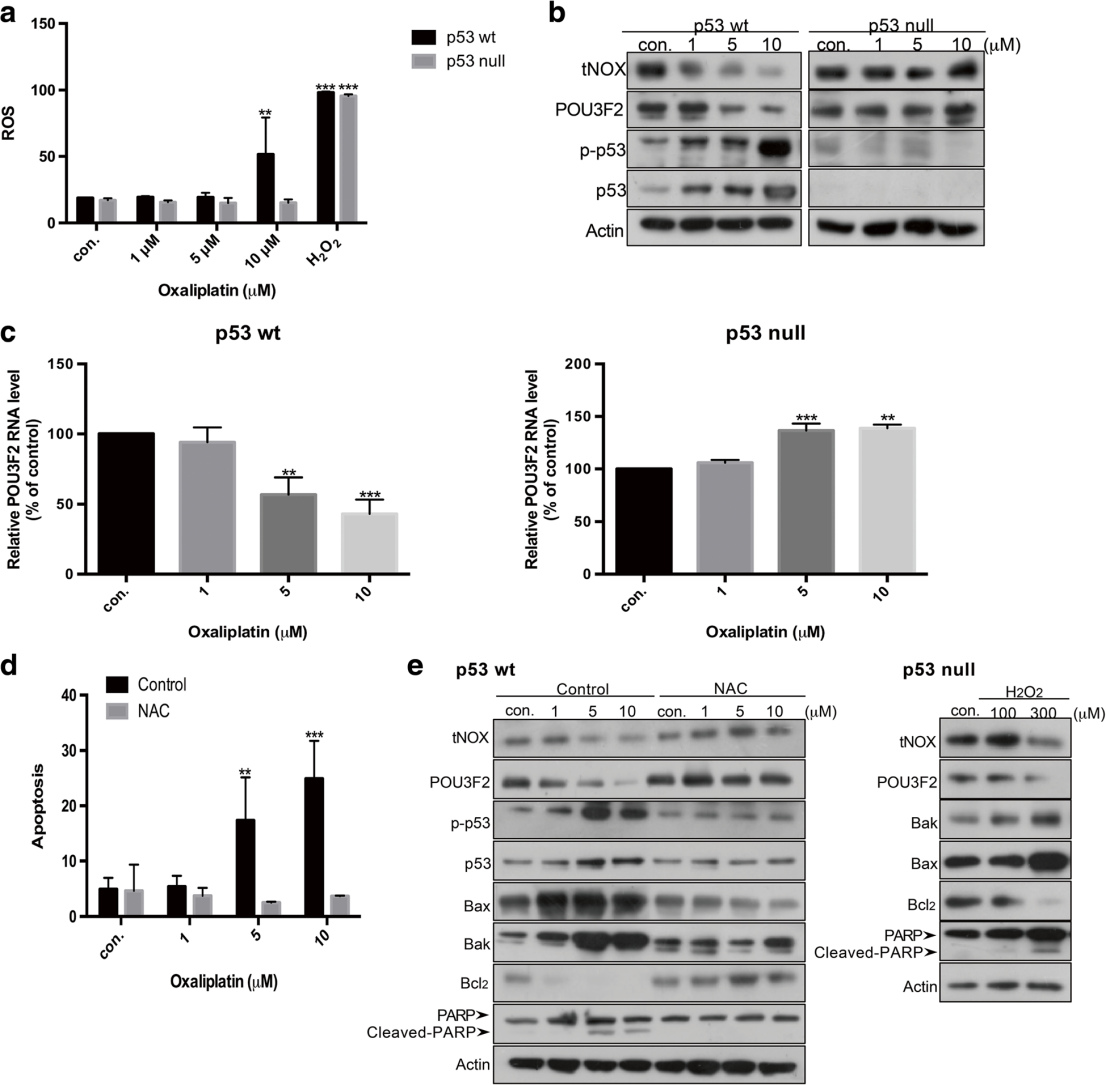
Oxaliplatin induces intracellular ROS generation, resulting in p53 phosphorylation, POU3F2 up-regulation, and apoptosis in p53-wild-type cells, but not in p53-null cells. **a**, The percent change in intracellular ROS generation was measured after cells were exposed to oxaliplatin for 1 h. **b**, Cells were treated with oxaliplatin for 24 h, and cell lysates were separated by SDS-PAGE and analyzed by Western blotting. β-Actin was used as an internal control. Representative images are shown. **c**, Cells were exposed to oxaliplatin and the RNA levels of POU3F2 were analyzed by RT-PCR. **d**, **e**, Cells were pre-incubated with or without 10 mM NAC at 37 °C for 2 h before exposure to oxaliplatin. The percentage of apoptotic cells was also determined by flow cytometry; the presented values (mean ± SEs) represent at least three independent experiments (**p < 0.01, ***p < 0.001 for treatments vs. controls) (**d**). Cell lysates were separated by SDS-PAGE and analyzed by Western blotting. β-Actin was used as an internal control. Representative images are shown (**e**)

In view of this significant correlation between POU3F2 and tNOX expression, we investigated the upstream signaling that modulates POU3F2 expression. The POU proteins comprise a large family of homeobox transcription factors binding to an octameric DNA sequence. Interestingly, p53 suppresses oncogenic activation through Brn-3a (POU4F1) [29], while Oct-1 (POU2F1) is known to be down-regulated by oxidative stress [30]. We thus examined whether ROS signaling and p53 are involved in the differential regulation of POU3F2 and subsequent tNOX expression in our system. A profound increase of ROS generation was elicited by 10 μM oxaliplatin in p53-wild-type cells, whereas no changes in ROS was observed in p53-null cells exposed to oxaliplatin (Fig. 7a). We also observed that the expression levels of tNOX and POU3F2 in p53-wild-type cells decreased as the oxaliplatin concentration increased, concurrent with enhancement of p53 phosphorylation, but no such changes was seen in p53-null cells (Fig. 7b). The observed enhancement of p53 phosphorylation prompted us to examine the transcriptional expression of POU3F2 in oxaliplatin-treated cells. Not surprisingly, we observed significant decreases and increases in the transcriptional expression levels of POU3F2 when we treated p53-wild-type and –null cells, respectively, with increasing concentrations of oxaliplatin (Fig. 7c). The activation of p53 seemed to correlate with oxidative stress. To examine whether such stress was essential in our system, we used N-acetyl cysteine (NAC) as a ROS scavenger. We found that NAC pre-treatment effectively reduced oxaliplatin-mediated apoptosis in p53-wild-type cells (Fig. 7d). Western blot analysis also revealed that the inhibition of ROS generation reversed the enhancement of phosphorylated p53, the down-regulation of POU3F2, tNOX, and Bcl2, the up-regulation of Bax and Bak, and the direct cleavage of PARP by caspase 3 (Fig. 7e). All of these findings support the notion that oxidative stress plays a vital role in oxaliplatin-mediated tNOX down-regulation and apoptosis in p53-wild-type cells. To validate that p53-null cells are capable of undergoing apoptosis, we used H_2_O_2_ to induce ROS in such cells. Indeed, ROS generation was associated with down-regulation of POU3F2, tNOX, and Bcl2, up-regulation of Bak and Bax, and enhanced PARP cleavage in p53-null cells (Fig. 7e), and these findings were accompanied by the induction of apoptosis (Fig. 7a).

**Fig. 8:**
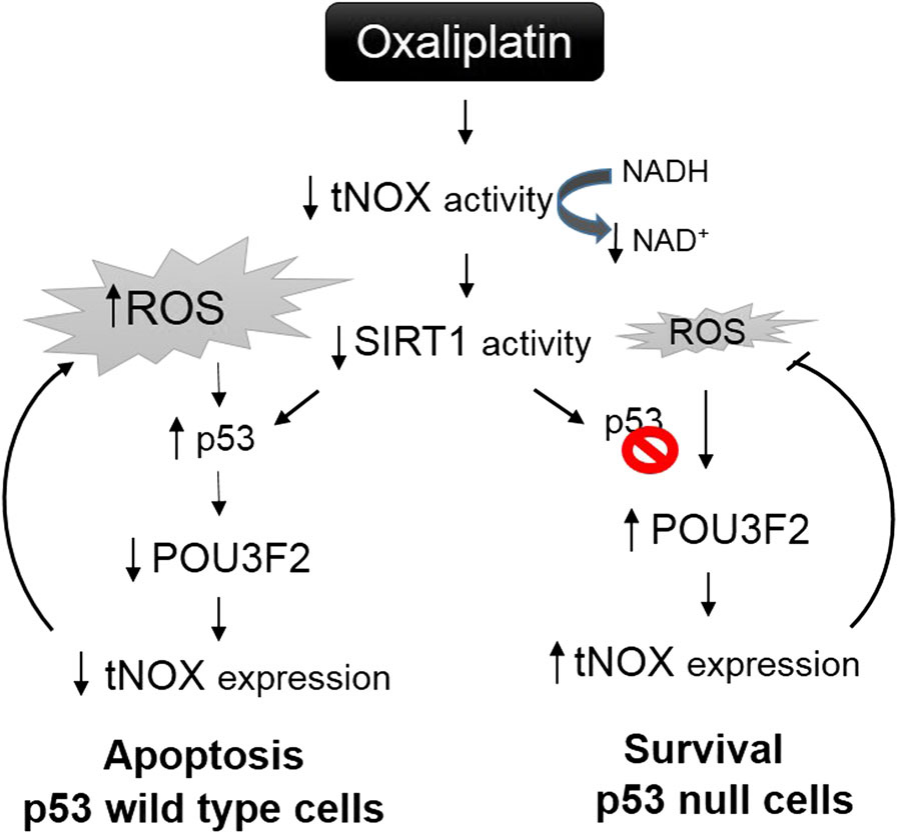
Schematic diagram of the pathways that lead to the differential responses of HCT116 p53-wild-type and p53-null cells toward oxaliplatin

Together, our various lines of evidence suggest that oxaliplatin treatment of p53-wild-type cells induced ROS generation, which attenuated POU3F2 and tNOX expression to diminish SIRT1, leading to apoptosis. In p53-null cells, in contrast, oxaliplatin initially inhibited tNOX activity, but the lack of p53 and a slight uptick in ROS generation prompted the up-regulation of POU3F2, leading to the transcriptional enhancement of tNOX expression and subsequent decreases in cellular and oxidative stress. Thus, the globally stable tNOX expression found in p53-null cells ultimately compensates for the inhibitory effect of oxaliplatin on tNOX activity, giving such cells a greater resistance against oxidative stress (Fig. 8).

## Discussion

The ultimate goal of cancer research is to discover ways to conquer cancer cells, such as by making them die selectively. In this study, we investigated the difference in cellular outcomes among oxaliplatin-treated colon cancer cells that differed in their p53 functionality. Importantly, we showed that ROS generation and p53 activation cooperatively act to inhibit tNOX and promote apoptosis in p53-wild-type colon cancer cells. The responsiveness to oxaliplatin was reflected in the ability of oxidative stress to down-regulate POU3F2 and tNOX, which appeared to lessen SIRT1 deacetylase activity, induce p53 activation, and trigger apoptosis in p53-wild-type cells. In contrast, p53-null cells treated with oxaliplatin failed to exhibit marked ROS generation, perhaps due to the up-regulation of tNOX; their higher oxidative stress tolerance and lack of p53 function led to enhanced cell survival. Notably, the depletion of tNOX could sensitize p53-null cells to both spontaneous and oxaliplatin-induced apoptosis. Our work thus clearly shows a scenario in which targeting of tNOX may be a potential strategy for cancer therapy in a p53-inactivated system.

To the best of our knowledge, this is the first study to suggest that ROS signaling and p53 collaboratively regulate POU3F2 and subsequent tNOX down-regulation. This is a milestone finding for both POU3F2 and tNOX. The molecular cloning of tNOX was initially reported in 2002 [31], but this is the first study to explore the regulation of its expression and that of its transcription factor, POU3F2. Members of the POU domain transcription factor family play essential roles in development and universally share the POU domain, which is a conserved motif that is important for DNA binding. However, each POU factor has its own unique expression pattern and function. Surprisingly, very few studies have focused on the correlation between oxidative stress and various POU factors. The exception is POU2F1, which was demonstrated to act as a sensor and modulate the expression of many target genes in response to cellular stress [32, 33]. Similarly, there is little literature examining the connection between p53 and POU factors. Early studies indicated that anti-oncogenic p53 suppresses the transcriptional expression of anti-apoptotic Bcl-2 and Bcl-x through POU4F1 (Brn-3a) [29, 34]. Most recently, p53 silencing was shown to upregulate POU2F1 to enhance CTHRC1 activation and Wnt-PCR signaling, and thus to support the metastasis of cervical cancer [35]. POU3F2, which was examined in the present study, belongs to the class III POU factors; also known as Brn2 or N-Oct3, it has been suggested to promote cell proliferation or even enhance metastatic potential [36–38]. GADD45 was identified as a p53-independent downstream target of POU3F2 in UVB-irradiated melanocytes [39], and we recently reported that POU3F2 is positively correlated with the transcriptional regulation of tNOX, potentially explaining the tumorigenic role of POU3F2 [28]. However, the upstream signaling involved in the regulation of POU3F2 remained largely unknown. In the present study, we dissected the involved pathways and reveal for the first time that ROS is an important upstream signaling component that acts via p53 activation to suppress POU3F2 expression, leading to the down-regulation of tNOX.

Another significant finding of the present work is our decoding of why oxaliplatin triggers differential cellular responses depending on the presence of p53. Oxaliplatin treatment is frequently associated with drug resistance; most such cases have lost p53 functionality, and tackling specific proteins, such as Nrf2, FOXM1/Mcl-1, and Oct4, has shown promise for combating this resistance [40–42]. As demonstrated in the present study, POU3F2-induced tNOX up-regulation and a lack of protein degradation contribute to the increased cancer cell survival seen in p53-null cells treated with oxaliplatin. We propose that the stably and enhanced expression of tNOX and consequently augmented ability to oxidize NADH may account for the ability of p53-null cells overcome oxidative stress. The feedback-mediated decrease in ROS generation and the lack of p53 activation lead to the up-regulation of POU3F2 and tNOX and subsequent increase in cancer cell survival. The role of tNOX in cell survival was further validated by our observation that the apoptotic populations was larger in tNOX-knockdown p53-null cells treated with oxaliplatin compared to the corresponding scrambled siRNA-transfected cells (Fig. 3). Conversely, accumulating data suggest that the cellular NAD^+^/NADH ratio is important for maintaining redox homeostasis, and that its disturbance by an excess of oxidizing or reducing equivalents is associated with stress [43, 44]. Decreased cellular NAD^+^ levels were previously associated with a higher susceptibility to oxidative stress [45]. This notion is also supported by our results demonstrating that, preferentially in p53-wild-type cells, oxaliplatin induced ROS generation and tNOX down-regulation to reduce the level of NAD^+^, and this virtuous cycle made the cells even more vulnerable to oxidative stress and ultimate apoptosis. Given that p53 is positioned at the frontier between cell survival and apoptosis, it is indeed a great leap forward to consider that targeting tNOX could be a strategy for managing cancer in a p53-defective system.

Target engagement is a key issue in the ability of anticancer drugs to exert the therapeutic effects. It is thus important to identify the functional site of a target protein. Researchers have faced many challenges and limitations in such work, but emerging methods enable target engagements to be determined in living systems [46]. We utilized one such method, the cellular thermal shift assay (CETSA), to show that oxaliplatin directly binds to tNOX and inhibits its oxidase activity. This notion was further supported by our observation that the intracellular NAD^+^/NADH ratio was reduced in oxaliplatin-treated p53-wild-type cells (Fig. 3). This reduction in the NAD^+^ level impacted SIRT1 activity to activate p53 and apoptosis. This is the first direct evidence that the oxaliplatin acts by engaging tNOX, as the previous studies used indirect methods, such as the investigation of downstream cellular responses to show the interaction. We observed an obvious shift in the thermal melting curves obtained from oxaliplatin-treated lysates of p53-wild-type cells compared to control lysates. Surprisingly, both cell lines exhibited similar increases in *T*_m_ following the addition of oxaliplatin. However, the p53-null cells failed to show the prominent sigmoid pattern evidenced by p53-wild-type cells. This indicates that different tNOX protein processing might occur in the presence or absence of functional p53. This could be an important pathway that is worthy of future investigation.

Taken together, our results show that oxaliplatin mediates differential cellular responses in colon cancer cells depending on their p53 status, and demonstrate that the ROS-p53 axis is important for regulating POU3F2 and its downstream target, tNOX, in this system. Furthermore, we used CETSA to show that tNOX directly engages with oxaliplatin in colon cancer cells lines, explaining its therapeutic action. These findings show that tNOX plays an essential function in the growth regulation and survival of cancer cells, and may provide a rational framework for the further development of tNOX inhibitors as a novel class of anticancer therapeutics.

## Conclusions

In conclusion, we focused on the p53 status and regulation of tNOX expression in HCT116 human colon cancer p53-wild-type and –null cells and show that ROS generation and p53 activation cooperatively act to inhibit POU3F2 and tNOX to promote apoptosis in p53-wild-type cells. In contrast, p53-null cells treated with oxaliplatin fail to exhibit marked ROS generation; their higher oxidative stress tolerance and lack of p53 function lead to enhanced cell survival (Fig. 8). Notably, the depletion of tNOX sensitizes p53-null cells to both spontaneous and oxaliplatin-induced apoptosis. Our work thus clearly shows a scenario in which targeting of tNOX may be a potential strategy for cancer therapy in a p53-inactivated system.

## Abbreviations

CETSA: Cellular thermal shift assay
CRC: colorectal cancer
RNAi: RNA interference
ROS: reactive oxygen species
SIRT1: Sirtuin 1
tNOX: tumor-associated NADH oxidase
